# Management of Penetrating Wrist Injuries in the Emergency Department

**Published:** 2009-11-02

**Authors:** Ioannis E. Bitzos, Mark S. Granick

**Affiliations:** Division of Plastic Surgery, New Jersey Medical School, University of Medicine and Dentistry of New Jersey, NJ 07103

## Abstract

**Objective:** Although several articles can be found on wrist trauma, a detailed literature search reveals limited articles that are focused specifically on penetrating puncture wounds of the wrist and the clinical approach to puncture-type penetrating wrist injuries. The number of penetrating injuries of the wrist has increased dramatically, especially since the widespread usage of the nail gun and staplegun technologies. The purpose of this article is not to analyze the approach to extensive wrist injuries and lacerations but how to evaluate and treat smaller puncture wounds that may potentially lead to significant clinical sequelae. **Methods:** This study describes a case of a staplegun injury to the radial artery of a 44-year-old man. The injury was treated with segmental arterial resection and anastomosis. **Results:** Postoperatively the patient had an uncomplicated course with good radial arterial flow and complete functional recovery of the hand. **Conclusion:** It is crucial to be conservative and have a high index of suspicion after puncture penetrating wrist injuries. The clinical presentation varies significantly according to the anatomical structure and there may be no obvious clinical signs of injury, as in the described case. In addition, even a minor deep foreign body in the wrist should alert one for immediate operative approach, possibly with the need for microsurgical preparedness for vessel or nerve repair. Only very superficial dermal or subcutaneous puncture wounds can be safely cleaned and managed in the emergency department.

## CASE REPORT

**Figure 1:**
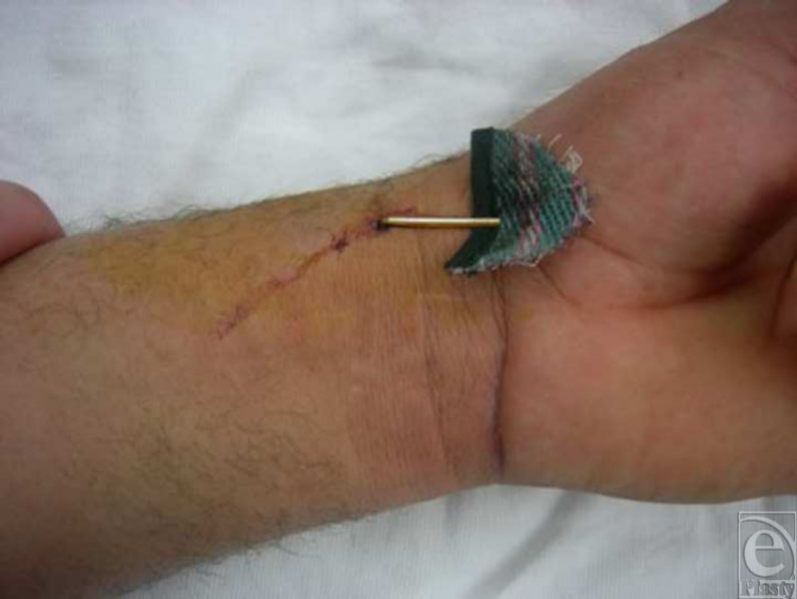
Gross appearance of staplegun injury.

**Figure 2:**
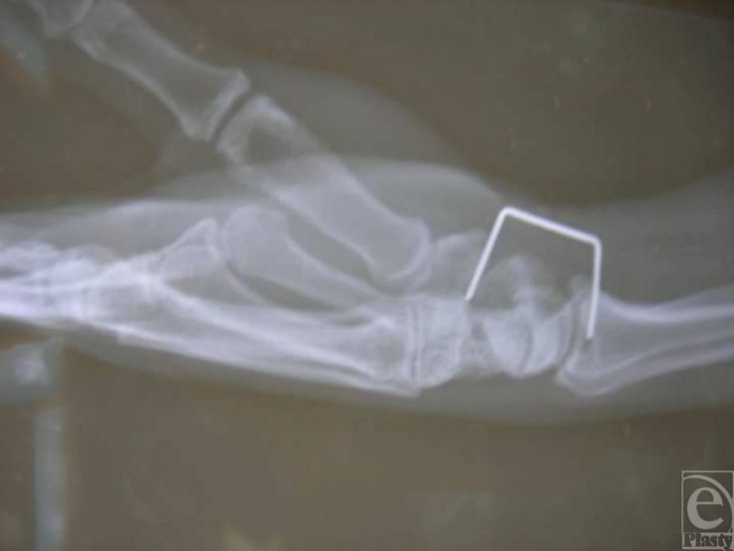
Radiologic appearance of staplegun injury.

A 44-year-old man, otherwise healthy, presented to the University Hospital emergency room, complaining of left wrist pain after an industrial type stapler was accidentally misfired in the volar-radial wrist area. Upon examination, there was a 2.5-cm rigid, nonmobile staple with one of the 2 staple insertion points corresponding to the anatomical position of the radial artery (Fig 1). However, the patient had an excellent radial pulse proximal and distal to the punctured spot. Allen's test was questionable. The other insertion point radiologically corresponded to the scaphotrapezial joint area (Fig 2). Clinically the patient had normal motor and sensory function of the entire hand and there was no hard evidence of vascular injury. However, although there was no bleeding from the wound, the decision was made to take the patient to the operating room since there was high suspicion for a radial artery injury based on the anatomical location of the staple. The patient was given adequate tetanus prophylaxis, antibiotics intravenously, and pain medications, and was taken to the operating room promptly.

**Figure 3:**
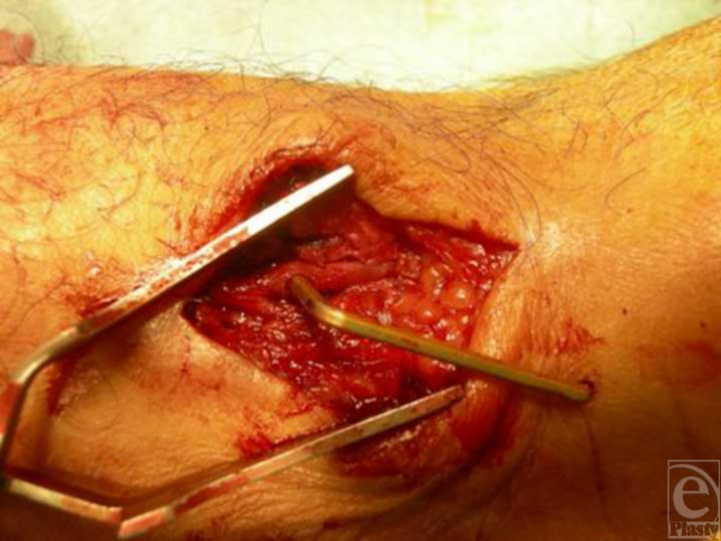
Through and through perforation of the radial artery with rigid fixation of staple on the radial bone.

**Figure 4:**
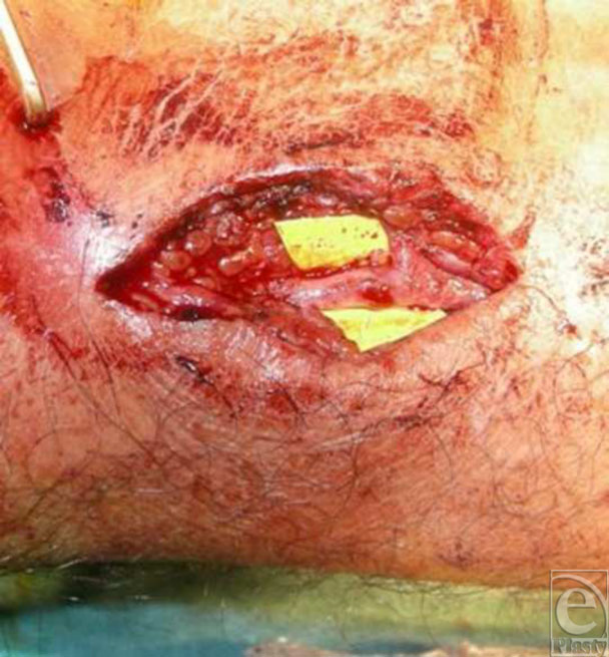
Microsurgical repair of the radial artery after segmental resection.

Intraoperatively an incision along the axis of the radius was performed over the potential radial artery injury site. The perforation across the radial artery was soon visualized (Fig 3). After adequate mobilization of the artery, the deeper course of the staple was visualized. There was no other structure involved and the radial artery was segmentally transected and anastomosed (Fig 4).

The wound was closed and an extension-blocking splint was applied. The patient had a 2+ radial pulse during the entire postoperative period and through follow-up. The patient underwent a short course of physical therapy and a postoperative angiogram was not performed as it would not change the clinical management.

## DISCUSSION

The unique anatomy of the volar wrist comes from the fact that in the small cross-sectional area of the volar wrist there are 12 tendons, 2 nerves and their branches, and 2 major arteries. A detailed history of the injury is important to diagnose retained foreign bodies in puncture wounds. Injuries are often more clinically significant on the volar surface, especially near the arterial conduits. In addition, the size of the radial artery varies and in the presented case it was particularly small (<2 mm). Allen's test is very important, but unfortunately not completely precise and reliable. Classic so-called “hard signs” of vascular injury include observed pulsatile bleeding, arterial thrill by manual palpation, bruit over or near the artery by auscultation, signs of distal ischemia, or visible expanding hematoma.

Factors that determine the degree of injury, the structures involved, and the overall prognosis are the size/diameter of the penetrated foreign body,^1^ its trajectory, presence of extensive lacerations, and bone age in case of bone fractures. Because of the number of osseous structures in the area, the potential joint spaces are multiple as are the potential infection sites. In the case of a radiopaque foreign body, radiologic findings can aid in the assessment of the anatomical structures involved and the planning of the operative exploration. Underpenetrated radiographs are the most readily available method for assessing wounds for retained foreign bodies. Radiopaque foreign bodies like metal, bone, or wood are visible in plain radiographs.^2^ Computed tomographic (CT) scanning is 100 times more sensitive in differentiating densities than plain radiography.^3^ However, CT scanning should be reserved for difficult cases when radiographs fail to show a foreign body, or the patient is at high risk for infection or joint involvement.^4^ Ultrasonographic sensitivity to foreign bodies ranges from 50% to 90%, and specificity ranges from 70% to 90%. Magnetic resonance imaging has not been assessed in this regard.^5^ In terms of the management, prompt exploration in the operating room is mandated in all cases of deep penetrating wrist trauma, if for no other reason, to debride the devitalized tissue (if any present) and to extensively irrigate the wound.

Penetrating wrist injuries, as compared to the more common sharp lacerations of the wrist, have frequently very few clinical signs of injury and especially if the foreign body has been removed, it may be impossible to adequately assess the depth and extent of the injury. It is important to remember that a sharp foreign body of even a very small diameter has the potential to seriously injure a wrist structure, especially if the foreign body is not fixed but is mobile. In these cases it can potentially cause a subcutaneous avulsion or laceration of a wrist structure while the external wound may be a small punctuate injury. Maintaining a stable position of the wrist during clinical and radiologic assessment in the emergency department is critical. Even in the cases of no obvious clinical signs of injury, blind removal of deep foreign bodies of the wrist is inappropriate and may potentially cause more injury to the underlying structures or delay and compromise operative exploration. Movement of the penetrated foreign body with movement of the digits most likely indicates tendon injury and patients should be advised not to move their hand and digits. If the point of penetration is corresponding to the area of the radial or ulnar pulses (or in a 2-cm radius around them), one must assume that the vessel is injured, regardless of the radiologic findings, and should plan for operative removal of the foreign body. Any sensory or motor deficit may signify that the foreign body transected or avulsed a nerve or nerve branch that may need to be repaired.

During exploration, planning of the incision is crucial and the incision should be performed so as to maximize the exposure to the potentially injured areas. The foreign body should be removed only after most, if not all, of the involved structures have been visualized and that is especially important for the vascular structures. If segmental vessel resection is deemed necessary, the surgeon needs to assess the defect length and plan for the possibility for need for a venous graft if the defect is long. The saphenous or smaller wrist veins are the most commonly used vein grafts. Loupe magnification or microsurgical techniques may be needed, especially if nerves are involved or if the vessel diameter is small. Tendons should be exposed and repaired as needed if their cross-sectional injury is significant or if the surgeon believes that it is indicated because of extensive tissue avulsion. Wound closure can be safely performed after extensive cleansing and irrigation, except if the wound is heavily contaminated. Most authorities recommend irrigating puncture wounds with normal saline at pressures of 5 to 8 psi to flush as much organic material, clothing, sand, and chemicals from the wound as possible. The authors prefer cadaveric dermis for temporary structure coverage in case of exposure and if there is a potential for desiccation. Any structural repair should be accompanied with application of a short arm splint, except for rare cases.

Hospitalization with administration of intravenous antibiotics is recommended in cases where structural repair or bone/joint injury occurred. Although infection is the most common complication of retained foreign bodies, the use of prophylactic antibiotics remains controversial. Most protocols recommend a first-generation cephalosporin with an aminoglycoside for highly contaminated wounds.^6^ However, recent evidence fails to support this practice.^7^ Tetanus prophylaxis is warranted for patients with these injuries. Potential complications in the early postoperative period include thenar or midpalmar infections, tenosynovitis, wound infection, osteomyelitis, anastomotic failure with need for re-exploration, or even hand loss if Allen's test finding was negative. Late complications include tendon adhesions, pseudoaneurysms,^8^ and thrombi/embolism. Frequent monitoring and vascular checks (eg, pulse presence, quality, and capillary refill) should continue for the first 24–48 hours. Consideration of anticoagulation and antiplatelet agents should be given, depending on individual surgeon's preference. Advising patients of the risks and symptoms of thrombosis or vascular occlusion is important so that they may quickly contact the surgeon or obtain evaluation in a local ED if problems occur.
